# Influence of submacular fluid on recovery of retinal function and structure after successful rhegmatogenous retinal reattachment

**DOI:** 10.1371/journal.pone.0218216

**Published:** 2019-07-03

**Authors:** Misato Kobayashi, Takeshi Iwase, Kentaro Yamamoto, Eimei Ra, Norifumi Hirata, Hiroko Terasaki

**Affiliations:** Department of Ophthalmology, Nagoya University Graduate School of Medicine, Nagoya, Japan; Massachusetts Eye & Ear Infirmary, Harvard Medical School, UNITED STATES

## Abstract

**Purpose:**

To determine the influence of residual submacular fluid (SMF) on the recovery of function and structure of the retina after successful rhegmatogenous retinal detachment (RRD) reattachment.

**Methods:**

We reviewed the medical records of all patients who had undergone successful RRD repair by scleral buckling (SB) surgery or by pars plana vitrectomy (PPV) from March 2011 to August 2014. Spectral-domain optical coherence tomographic images of the macular regions were used at 1, 2, 3, 6, 9, and 12 months following the surgery. The best-corrected visual acuities (BCVA) were evaluated at the same times.

**Results:**

The eyes with a macula-off RRD that were treated by SB surgery had a significant higher incidence of residual SMF (52%) than those treated by PPV (6.8%; *P* <0.001). Nevertheless, the postoperative BCVA was significantly improved in the eyes that had undergone SB surgery (*P* = 0.007). The postoperative BCVAs were not significantly different between the groups in which the SMF was absorbed (12 eyes) and not absorbed (13 eyes) within 1 month after the SB surgery. The photoreceptor outer segment length and the presence of a foveal bulge were not significantly different between these two groups at 12 months. Multiple regression analyses showed that the presence of a foveal bulge (*β* = 0.531, *P* = 0.001) and the duration of the retinal detachment before surgery (*β* = 0.465, *P* = 0.002) but not the duration of the SMF were independent factors significantly correlated with the final BCVA.

**Conclusions:**

These results suggest that the postoperative residual SMF does not significantly disrupt the functional and structural recovery of eyes with macula-off RRD treated by SB surgery.

## Introduction

A rhegmatogenous retinal detachment (RRD) is a common cause of visual impairments, and the main treatment for a RRD is a surgical reattachment of the retina [1]. There are several types of retinal reattachment surgery, e.g., scleral buckling (SB), pneumatic retinopexy, and pars plana vitrectomy (PPV) combined with or without SB surgery [2]. The choice of which patients will benefit from SB or PPV for the primary RRD repair is generally decided by the preoperative findings and the surgeons’ preferences [3].

With the recent advancements in the vitrectomy instruments and techniques, PPV has become the first choice for many surgeons especially for patients with pseudophakic RRD [2]. On the other hand, SB surgery is preferred for younger patients with RRD associated with an atrophic retinal holes [4].

Residual submacular fluid (SMF) is frequently found after successful SB surgery for macula-off RRD even when the retina appears to be fully attached by ophthalmoscopy, and all retinal breaks appear to be adequately sealed. Machemer reported this phenomenon when he described small collections of SMF after the resolution of experimental retinal detachment in owl monkeys [5]. Recent improvements in the resolution of spectral-domain (SD) optical coherence tomographic (OCT) instruments have made it possible to obtain more precise and accurate images of the foveal microstructures, and the higher resolution images have allowed clinicians to detect subtle SMF more easily and accurately. Studies have reported a delayed absorption of SMF following SB surgery in humans [6, 7].

However, it remains unclear whether the visual outcomes are affected by the residual SMF; some authors have suggested that persistent SMF can influence visual outcome [8–10], while others have stated there was no influence[11–13]. These contradictory conclusions may have been caused by the short follow-up periods, small case numbers, or other unexamined factors.

The length of the photoreceptors has been shown to be reduced in cases of macula-off RRD in which there is a separation of the macular region of the retina from the retinal pigment epithelium (RPE) [14]. It has been reported that the photoreceptors were shorter in eyes affected by macula-off RRD than that of the fellow unaffected eyes at 1 month after successful reattachment [15, 16]. After reattachment, the length of the photoreceptors increased significantly in parallel with the improvement of the best-corrected visual acuity (BCVA) [17]. Careful examinations of the SD-OCT images of normal eyes shows a bulging of the ellipsoid zone (EZ) in the central fovea, termed the foveal bulge. Recent OCT studies have shown that the presence or absence of a foveal bulge was significantly correlated with the BCVA in eyes with retinal diseases [18–21] including that after successful reattachment of a RRD [17] [22].

Yet, it is still not determined whether the photoreceptors damaged by the RD can recover after successful retinal reattachment in the presence of residual SMF in eyes with macula-off RRD. In addition, it remains undetermined whether the SMF affects the BCVA and the formation of the foveal bulge.

Thus, the purpose of this study was to determine the influence of residual SMF on the recovery of the function and structure of the photoreceptors after a reattachment of a RRD. To accomplish this, we measured the BCVA and examined the SD-OCT images before and after successful retinal reattachment in eyes with macula-off RRD that had undergone either SB surgery or PPV.

## Patients and methods

### Ethics statement

This was a retrospective, observational, comparative, single-center study, and the procedures used conformed to the Declaration of Helsinki and were approved by the Institutional Review Board and Ethics Committee of the Nagoya University Graduate School of Medicine. Written informed consent was obtained from all patients.

### Subjects

We reviewed the medical records of all patients who had undergone successful RRD repair by SB surgery or by PPV at the Nagoya University Hospital from March 2011 to August 2014. All patients had comprehensive ophthalmic examinations including measurements of the BCVA, intraocular pressure, and axial length. They also had slit-lamp, ophthalmoscopic, and SD-OCT examinations at 1, 2, 3, 6, 9, and 12 months following the surgery. The definition of the duration of the macula detachment was the interval between the beginning of the symptom and the time of the surgery. The Snellen visual acuity values were converted to the logarithm of the minimum angle of resolution (logMAR) units to create a linear scale for the statistical analyses.

The patients with macula-off RRD were divided into two groups according to the presence or absence of SMF as determined by examination of the SD-OCT images at one month following the surgery. The eyes were also divided into those with rapid SMF resolution group in which the SMF was resolved within 1 month, and the persistent SMF group in which the SMF was present at one month following the RRD surgery.

### Measurements using optical coherence tomographic images

The Spectralis OCT instrument was used to obtain the SD-OCT images. The horizontal cross-sectional images recorded at each visit after successful retinal reattachment were analyzed. The thickness of the retinal layers was measured on the same selected central foveal scan using the caliper measurement tool of the SD-OCT instrument. The external limiting membrane (ELM)-ellipsoid zone (EZ) thickness was defined as the distance between the outer border of the ELM to the outer border of the EZ. The EZ-RPE thickness was defined as the distance between the outer border of EZ to the inner border of the RPE. The thickness of the retinal layers was measured manually by 2 operators masked to all information including the pre-operative condition of the retina.

A foveal bulge was defined as an EZ-RPE thickness at the central fovea that was >10 μm greater than the average EZ-RPE thickness at 250 μm temporal and nasal to the central fovea. The degree of agreements between the two operators for the presence of a foveal bulge was determined by intraclass correlation coefficient (ICC).

### Surgical techniques

The surgery was performed under retrobulbar anesthesia with 2.5 mL of 2% lidocaine and 2.5 mL of 0.5% bupivacaine. Basically, the SB surgery was performed for retinal detachments secondary to atrophic round retinal hole(s), and the PPV procedure was performed for posterior vitreous detachment (PVD)-related retinal detachments.

In the SB surgery, all of the retinal breaks were identified in all patients and were treated by transscleral cryotherapy. Mattress sutures were placed 7.0 to 7.5 mm apart with 4–0 supramid (Kono, Chiba, Japan) for the circumferential segmental buckle, and a silicone sponge (Mira No. 506; Mira, Inc, Waltham, MA) was sutured as an explant in all cases. Scleral dissection and extraocular muscle disinsertion were not required for any patients. Subretinal fluid drainage was performed if necessary. An intraocular tamponade was not used in all cases. Dexamethasone (MSD K.K., Tokyo, Japan) was injected subconjunctivally at the end of surgery. No intraoperative complications were encountered. Reattachment of the retina was achieved in all patients after the initial surgery.

To begin the PPV procedure, a trocar was inserted at an approximate angle of 30° parallel to the limbus. Once the trocar was past the trocar sleeve, the angle was changed to be perpendicular to the retinal surface. After creating the three ports, PPV was performed using the Constellation system (Alcon Laboratories, Inc., Fort Worth, TX). The vitreous was removed as completely as possible without the use of intravitreal triamcinolone, then fluid-air exchange and subretinal fluid drainage from the causative retinal tear(s) or iatrogenic hole were performed, but perfluorocarbon was not used for the drainage of SMF. Endo-photocoagulation was applied to the causative retinal tear(s) or any iatrogenic holes. Then 20% sulfur hexafluoride (SF_6_) was injected into the vitreous upon completion of the PPV. After the IOP was adjusted to normal levels, the cannulas were withdrawn.

### Exclusion criteria

Eyes were excluded if they had had encircling procedures, dense ocular media (e.g., vitreous hemorrhage, vitreous opacity), preexisting macular conditions (e.g., macular hole, vascular occlusive diseases, or diabetic retinopathy), proliferative vitreoretinopathy (PVR) = grade C [23], and clinical evidence for postoperative changes likely to interfere with an accurate evaluation of the retinal layers, e.g., recurrent RRD, epiretinal membrane, or cystoid macular edema.

### Statistical analyses

The values are presented as the means ± standard deviations (SDs). Independent *t*-tests were used to determine the significance of differences in normally distributed data, and chi-square tests were used for categorical data. One-way analysis of variance (ANOVA) was used to evaluate the changes in the BCVA, and Pearson’s correlation coefficient tests were used to determine the significance of the differences between them. Multiple linear regression analyses were used to determine the significance of the association between final BCVA and independent variables. A *P* value <0.05 was considered statistically significant.

## Results

### Demographics and surgical parameters of patients

One hundred and ninety-nine eyes of 192 patients with a RRD were studied. Eighty-one eyes of 77 patients underwent SB surgery with or without an encircling band, and 118 eyes of 115 patients underwent PPV in the Department of Ophthalmology, Nagoya University for the repair of RRD from March 2011 to August 2014. Of these, 120 eyes of 115 patients had macula-off RRD and 79 eyes of 77 patients had macula-on RRD. A total of 66 eyes with macula-off RRD was excluded; 6 for concomitant encircling procedures, 5 for the presence of PVR grade C, 5 for the presence of vitreous hemorrhage, 1 for the presence of macular hole, 2 for the presence of diabetic retinopathy,2 for the presence of postoperative development of dense cataract, 5 for the presence of macular edema, 3 for the presence of a recurrent RRD, 1 for the presence of a significant epiretinal membrane during the follow-up period, and 36 for an inability to attend regular follow-up examinations. In the end, 25 eyes with macula-off RRD that had undergone SB surgery and 29 eyes with macula-off RRD that had undergone PPV were studied (Table 1).

**Table 1:** Patient clinical characteristics.

Characteristic	Scleral buckling	Vitrectomy	P value
n	25	29	-
Age (years)	40.5 ± 15.6	59.6 ± 12.0	< 0.001
Sex (male/female)	19/6	18/11	0.276
Initial BCVA (logMAR)	0.77 ± 0.68	1.12 ± 0.73	0.056
Final BCVA (logMAR)	0.15 ± 0.24	0.24 ± 0.24	0.234
Axial length (mm)	25.8 ± 1.6	25.5 ± 1.8	0.707
Rapid resolution of SMF/Persistent SMF	12/13	27/2	< 0.001
ELM-EZ thickness at Month 12 (μm)	31.72 ± 3.41	31.82 ± 3.15	0.887
EZ-RPE thickness at Month 12 (μm)	43.04 ± 7.06	42.21 ± 5.51	0.721

BCVA: best-corrected visual acuity, logMAR: logarithm of the minimum angle of resolution scale, SMF: submacular fluid

In the eyes with macula-off RRD, only two eyes (6.8%) had residual SMF of the 29 eyes treated by PPV at one month following the surgery, and 13 eyes (52%) had residual SMF of the 25 eyes treated by SB surgery at one month following surgery (*P* <0.001). The demographics and surgical parameters in the rapid SMF resolution group and the persistent SMF group are shown in Table 2. The presence of a grade B PVR was detected in 2 eyes in the PPV with rapid SMF resolution group. The repeatability of the measurements between the graders was good with an ICC of 0.95 for the ELM- EZ thickness, 0.95 for the EZ-RPE thickness, and 0.96 for the presence of foveal bulge.

**Table 2:** Characteristics in rapid resolution and persistent SMF group.

Characteristic	Scleral buckling			Vitrectomy	
	Rapid resolution	Persistent SMF	P value	Rapid resolution	Persistent SMF
n	12	13	-	27	2
Age (years)	40.17 ± 15.2	40.77 ± 16.6	0.926	59.59 ± 12.2	59.00 ± 11.3
Sex (male/female)	10/2	9/4	0.419	17/10	1/1
Initial BCVA (log MAR)	1.06 ± 0.81	0.50 ± 0.40	0.037	1.15 ± 0.74	0.70 ± 0.25
Final BCVA (log MAR)	0.17 ± 0.27	0.14 ± 0.23	0.770	0.24 ± 0.24	0.20 ± 0.28
Duration of macula detachment (day)	7.8 ± 10.9	15.1 ± 13.4	0.155	4.82 ± 4.93	2.50 ± 0.71
Axial length (mm)	25.8 ± 2.0	25.7 ± 0.9	0.846	25.6 ± 1.6	25.1 ± 0.8
Extent of RD (clock hour)	5.25 ± 1.35	4.85 ± 1.67	0.589	6.33 ± 1.33	7.00 ± 2.83
Location of RD (superior / inferior)	8/4	5/8	0.167	21/6	2/0
Extent of quadrant-wise buckle (degrees)	89.2 ± 26.8	95.0 ± 31.1	0.621		
Drainage of subretinal fluid (+/-)	10//2	9/4	0.409	27/0	2/0
Presence of foveal bulge (+/-)	8/4	9/4	0.893	17/10	1/1
ELM-EZ thickness at Month 12 (μm)	30.83 ± 1.89	32.54 ± 4.29	0.218	31.85 ± 3.24	31.50 ± 2.12
EZ-RPE thickness at Month 12 (μm)	42.33 ± 7.08	43.69 ± 7.25	0.640	42.4 ± 5.6	39.5 ± 3.5

SMF: submacular fluid, logMAR: logarithm of the minimum angle of resolution scale, BCVA: best-corrected visual acuity, RD: retinal detachment

### Changes of BCVA after surgery for macula-off RRD treated by SB surgery

**Fig 1:**
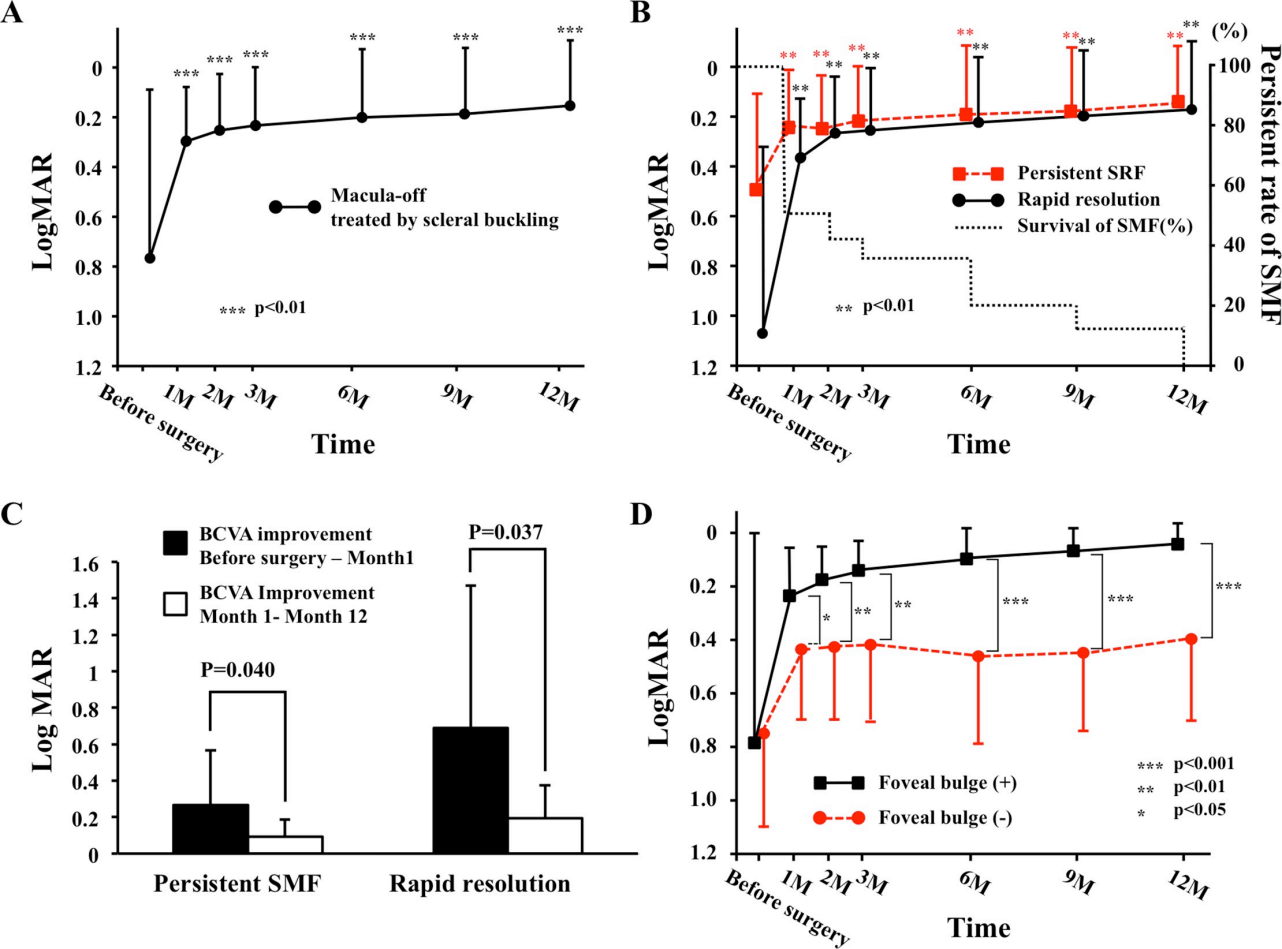
Changes in the mean best-corrected visual acuity (BCVA) after scleral buckling (SB) surgery in eyes with macula-off rhegmatogenous retinal detachment (RRD). **The persistence rate of the submacular fluid (SMF) is also shown.** The BCVA significantly improved in both of the rapid resolution SMF group and the persistent SMF group following surgery. The mean pre-operative BCVA in the residual SMF group was significantly better than that in the rapid SMF resolution group (*P* = 0.037, Table 2), but the BCVAs did not differ significantly at any postoperative times (B). The improvement of the BCVA from preoperative to Month 1 was significantly greater than that from Month 1 to Month 12 in both the persistent SMF group (*P* = 0.040) and the rapid SMF resolution group (*P* = 0.037) (C). The mean postoperative BCVA in eyes with a foveal bulge was significantly better in eyes without a foveal bulge throughout the postoperative follow-up period (D).

The BCVAs in eyes macula-off RRD treated by SB surgery are shown in Fig 1. The BCVA significantly improved during the follow-up period *(P <*0.001).

The postoperative BCVA was significantly improved in both groups (*P* <0.001, *P* = 0.015, respectively), and it was significantly improved even at 1 month compared with the preoperative BCVA in both groups (*P* = 0.009, *P* = 0.007, respectively). In addition, the improvement of the BCVA between the preoperative value and that at 1 month was greater than that between 1 and 12 months for both the rapid resolution SMF group (*P* = 0.037) and the persistent SMF group (*P* = 0.040).

**Fig 2:**
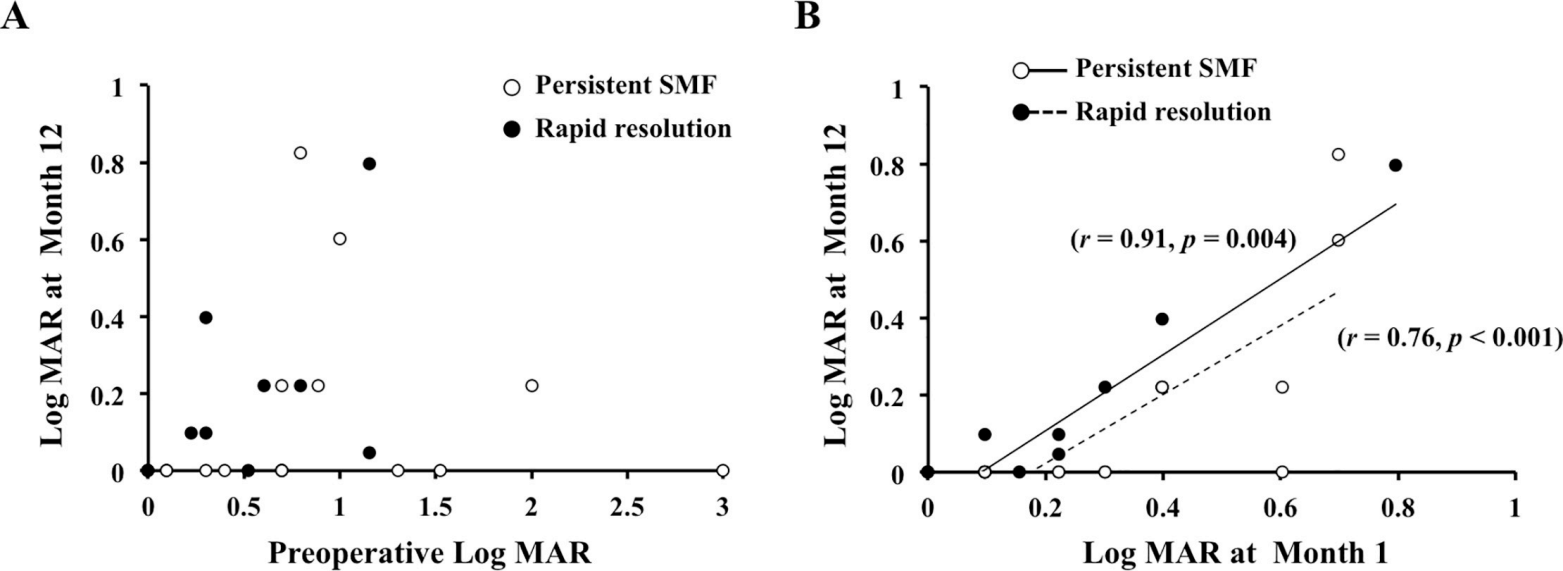
Correlations between pre- and post-operative visual acuity. There was no significant correlation between the preoperative BCVA and the final BCVA (A), but the BCVA at 1 month after surgery was significantly correlated with the final BCVA in both the rapid SMF resolution group (r = 0.76, *P* = 0.004) and the persistent SMF group (r = 0.91, *P* <0.001) (B).

The BCVA at 1 month after the SB surgery was significantly correlated with the final BCVA in the rapid SMF resolution group (*r* = 0.76, *P* = 0.004) and also in the persistent SMF group (*r* = 0.91, *P* <0.001). However, the difference in the pre- and postoperative BCVA was not significantly correlated in the both groups (Fig 2).

### Correlations between retinal microstructures and BCVA in macula-off group

**Fig 3:**
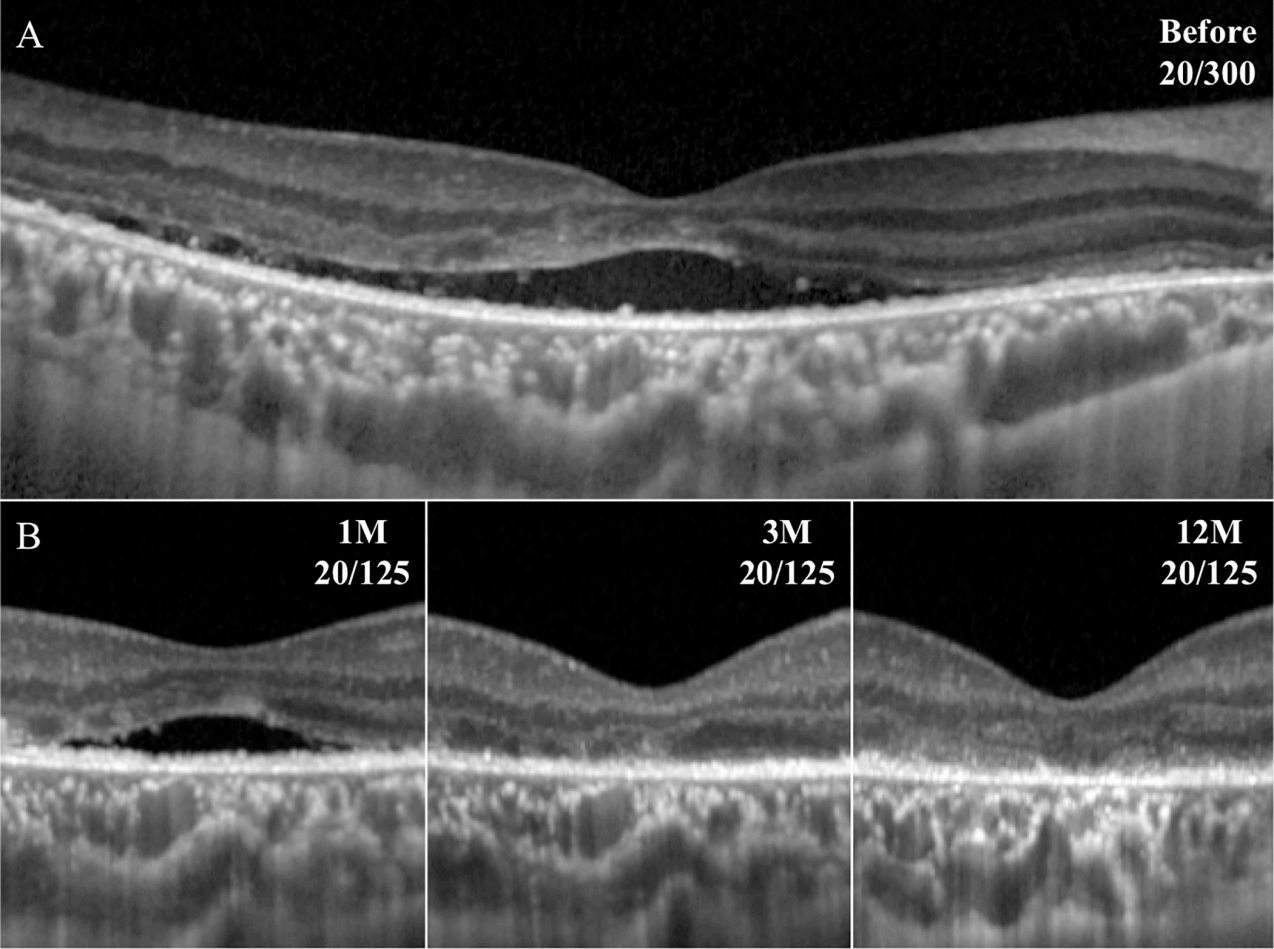
Representative SD-OCT images in eye with macula-off RRD. The SMF remained at 1 month but was not detected at 3 months. However, the ELM, the EZ, and the CIZ appear to be fragmented and thin even after the resolution of the SMF.

**Fig 4:**
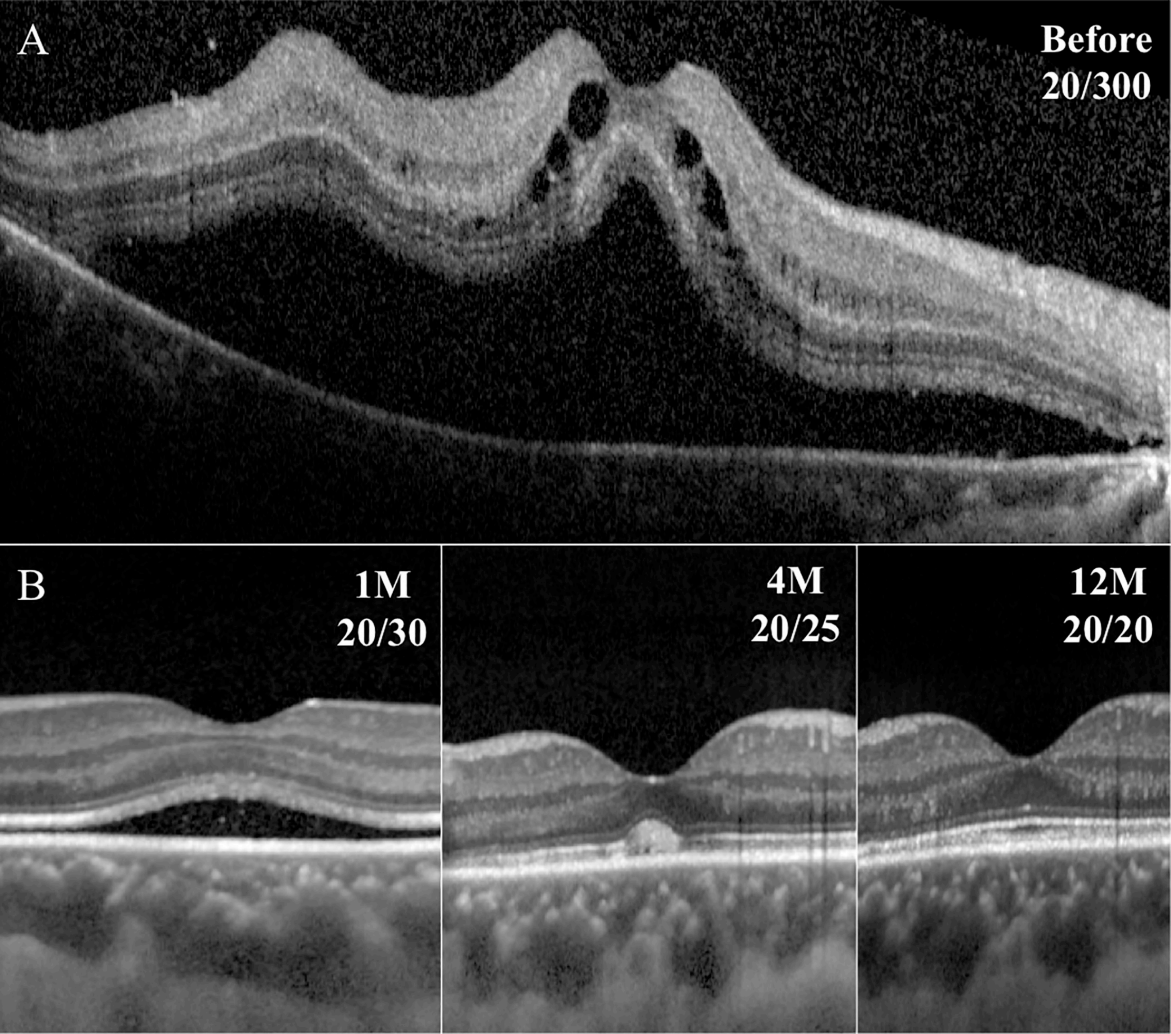
Representative SD-OCT images of eye with macula-off RRD. The SMF remained until month 4, but the ELM, EZ, and CIZ are present but the foveal bulge is not detected.

**Fig 5:**
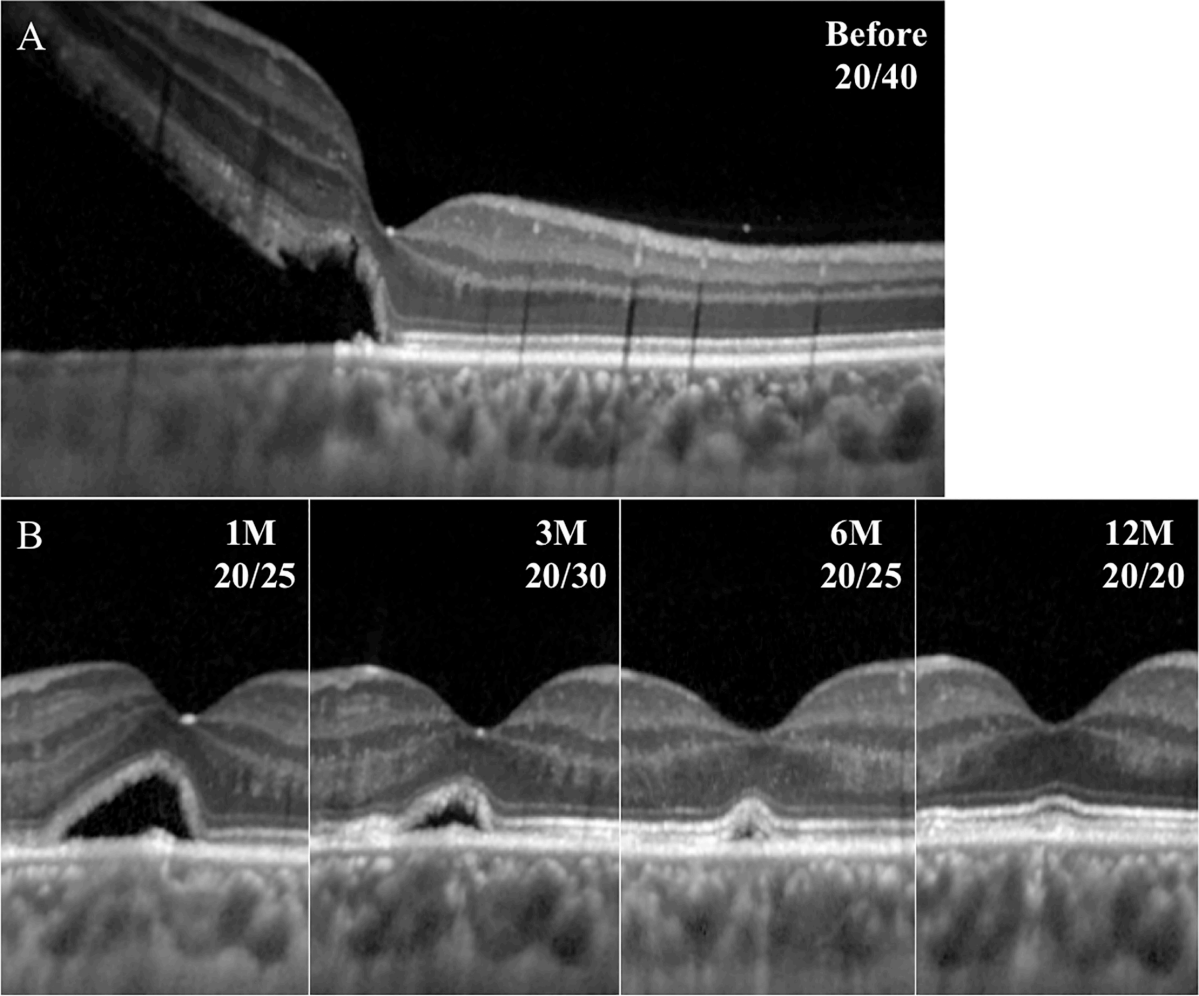
Representative SD-OCT images in eye with macula-off RRD. The SMF remained until month 6, and the foveal bulge and the outer 3 retinal layers are present.

The difference in the ELM-EZ and the EZ-RPE thicknesses at 12 months after the surgery between the rapid resolution and the persistent SMF group was not significant (Table 2). The EZ-RPE thickness was significantly correlated with the final vision in the macula-off RRD treated by SB surgery (*P* = 0.001), but the ELM-EZ thickness was not significantly correlated with the final BCVA (Figs 3, 4 and 5).

A foveal bulge was observed in 17 eyes in the macula-off RRD group treated by SB surgery: 8 eyes in the rapid SMF resolution group and 9 eyes in the persistent SMF group. The differences in the age, sex, duration of macular detachment, preoperative BCVA, and axial length were not significant between patients with and without the presence of a foveal bulge. The mean postoperative BCVA in eyes with a foveal bulge was significantly better in eyes without a foveal bulge throughout the postoperative follow-up period (Table 3).

**Table 3:** The mean BCVA in foveal bulge(+) and (-) group.

Group	Before surgery	Month 1	Month 2	Month 3	Month 6	Month 9	Month 12
Foveal bulge(+)	0.81 ± 0.81	0.24 ± 0.18	0.16 ± 0.11	0.14 ± 0.11	0.11 ± 0.12	0.06 ± 0.09	0.05 ± 0.08
Foveal bulge (-)	0.76 ± 0.38	0.44 ± 0.27	0.43 ± 0.28	0.42 ± 0.29	0.46 ± 0.34	0.45 ± 0.30	0.40 ± 0.31
P-value	0.827	0.040	0.002	0.003	<0.001	<0.001	<0.001

BCVA: best-corrected visual acuity

Multiple stepwise regression analyses showed that the SB and PPV surgical procedures (*β* = 0.596, *P* <0.001) and the status of the macula, on or off, (*β* = 0.527, *P* <0.001) were independent factors significantly correlated with the eyes with residual SMF at 1 month following the surgery (Table 4). In addition, the presence of a foveal bulge (*β* = 0.531, *P* = 0.001) and the duration of the retinal detachment (*β* = 0. 465, *P* = 0.002) were independent factors significantly correlated with the final BCVA (Table 5), and the preoperative BCVA (*β* = -0.945, *P* = 0.001) and the age (*β* = 0. 153, *P* = 0.017) were independent factors significantly correlated with the improvement of BCVA (Table 6)

**Table 4:** Results of multiple stepwise regression analysis for independence of factors contributing to disappearance of SMF.

Variable				
Dependent	Independent	*β*	VIF	*p*-value
Appearance of SMF	Duration of macular detachment	0.293	1.000	0.155
	Location of RD	-0.214	1.097	0.316
	Extent of RD	-0.189	1.028	0.360
	Age	0.185	1.242	0.417
	Sex	0.150	1.185	0.501
	Drainage of subretinal fluid	-0.034	1.289	0.885
	Axial length	0.009	1.027	0.966

VIF: variance inflation factors, BCVA: best-corrected visual acuity, SMF: submacular fluid, RD: retinal detachment

**Table 5:** Results of multiple stepwise regression analysis for independence of factors contributing to final vision.

Variable				
Dependent	Independent	*β*	VIF	*p*-value
Final BCVA	Presence of foveal bulge	0.531	1.231	0.001
	Duration of macular detachment	0.465	1.231	0.002
	Axial length	0.157	1.105	0.215
	Age	-0.145	1.008	0.304
	Preoperative BCVA	0.074	1.002	0.543
	Duration of SMF appearance	-0.037	1.002	0.762
	Sex	-0.018	1.003	0.896

VIF: variance inflation factors, BCVA: best-corrected visual acuity, SMF: submacular fluid

**Table 6:** Results of multiple stepwise regression analysis for independence of factors contributing to the improvement of vision.

Variable				
Dependent	Independent	*β*	VIF	*p*-value
Improvement of BCVA	Preoperative BCVA	-0.945	1.063	<0.001
	Age	0.153	1.063	0.017
	Presence of foveal bulge	-0.075	1.370	0.174
	Duration of SMF appearance	-0.084	1.954	0.221
	Sex	0.031	1.499	0.753
	Duration of macular detachment	-0.017	1.226	0.771
	Axial length	0.001	1.043	0.994

VIF: variance inflation factors, BCVA: best-corrected visual acuity, SMF: submacular fluid

## Discussion

A significantly higher number of eyes with residual SMF was observed in the macula-off RRD treated by SB surgery than in eyes treated by PPV. There was no significant difference in any factors including the final BCVA between the persistent SMF group and the rapid SMF resolution group. The multivariate regression analyses showed that presence of a foveal bulge and the duration of the retinal detachment before surgery, but not the duration of the residual SMF, were independent factors significantly correlated with the final BCVA.

Our results showed that a significantly higher number of eyes with residual SMF was detected in the macula-off RRD treated by SB surgery than that in eyes treated by PPV. These results are consistent with several reports that the absorption of the SMF tended to be more delayed following SB surgery than after PPV [6, 7, 24]. This might be because the type of surgery affects the make-up of the residual SMF. However, we performed SB surgery on RRDs secondary to atrophic round retinal hole(s), and PPV for PVD-related RRDs. This would suggest that the preoperative characteristics of the SMF were different between the SB and the PPV treated groups.

In earlier studies, the incidence of residual SMF after SB surgery for primary RRD ranged widely from 9 to 94% [9, 10, 13, 25–27]. This variation among studies is probably related to differences in study design, baseline characteristics of the patients, and the operative procedures. The results of our study showed that 52% of the eyes with macula-off RRD had residual SMF in the OCT images at 1 month after the SB surgery which is consistent with most previous reports.

Benson et al reported that the incidence and patterns of SMF after SB surgery in a prospective study [8]. They also investigated the association between the presence of SMF in the OCT images at 6 weeks and some clinical factors, such as age, sex, refractive status, RD type, clock hours of buckle, duration of RD, presence of outer retinal wrinkling on OCT, postoperative posture, and drainage of SRF at surgery. They reported no significant associations among these parameters [8]. Also, our results showed no association between the presence of SMF and any clinical factors including the duration of the macular detachment, location and extent of the RD, and with or without of drainage of the subretinal fluid.

On the other hand, it has been suggested that a younger age, phakia, and longer standing detachment as possible preoperative risk factors for persistent SMF after SB surgery [28]. Our results showed that the mean age of the eyes treated by SB surgery was significantly younger than that in eyes treated by PPV. In eyes with atrophic round retinal hole(s), a smaller volume of vitreous humor would tend to flow into the subretinal space because the age-related liquefaction of the vitreous body had not progressed. This will result in a slower progression of the detachment, and should lead to RRD eyes with atrophic round retinal hole(s) to have a systematic chronicity and the fluid current induced differences in the viscosity, protein, and cellular content of the subretinal fluid [29]. This would explain our observation of a higher incidence of SMF in the macula-off RRD treated by SB surgery.

Our results showed no significant differences in the variables except for the preoperative vision between the rapid SMF absorption group and the persistent SMF group. It is also possible that the characteristics of the SMF is different before the surgery. The residual SMF has been reported to be viscous with high level of cellularity [28]. Veckeneer et al found that subretinal fluid samples collected during RRD surgery had a high concentration of rhodopsin-containing cells, and they hypothesized that the residual SMF occurred because of the fluid composition in relation to the absence of a PVD and the chronicity of the detachment [28]. Accordingly, the long persistent SMF group may have higher viscosity SMF before the surgery.

Yet, it is still controversial whether the residual SMF affects the postoperative recovery of the BCVA after successful reattachment with SB surgery [8–13]. Our results showed that there was no significant difference in the final BCVA between the rapid SMF absorption group and the persistent SMF group in eyes with macula-off RRD treated by SB surgery. In addition, multiple stepwise regression analyses showed that there was no association between the final BCVA or the improvement of BCVA and the duration of SMF appearance. These results suggest that the residual SMF would not affect the recovery of the BCVA which is in keeping with previous reports [11–13].

The BCVA at 1 month was strongly and positively correlated with that at 12 months but the preoperative BCVA was not significantly correlated with the BCVA at 12 months in both groups. In addition, the improvement of the BCVA between the preoperative and at 1 month, when the SMF was completely absorbed, was greater than that between 1 month and 12 months in the rapid SMF absorption group. Interestingly, the improvement of the BCVA between the preoperative and at 1 month, when the SMF still remained, was greater than that between 1 month and 12 months in the persistent SMF group. These results indicate that vision can be significantly improved while the SMF remained. In addition, it suggests that the closure of the retinal break(s) which prevented vitreous humor from flowing into the subretinal space, might be more important than the absorption of the SMF in terms of improving the BCVA and recovery of the photoreceptors.

Morphologically, there was no significant difference in the final BCVA and the thickness of ELM-EZ (photoreceptor inner segment thickness) and the EZ-RPE thickness (outer segment thickness) between the rapid SMF absorption group and the persistent SMF group in eyes with macula-off RRD treated by SB surgery. The foveal bulge was formed until 12 months even in the persistent SMF group after the absorption of the SMF. In addition, there was no significant difference in the ratio of forming the foveal bulge between the rapid absorption group and the persistent SMF group. It has been reported that vision in eyes with a foveal bulge is significantly better than without a foveal bulge [17, 22]. Our results also showed that eyes with a foveal bulge had significantly better BCVA than eyes without a foveal bulge throughout the follow-up period after SB surgery. The formation of the foveal bulge is believed to be due to an increase in the cone density during the recovery of the fovea in eyes following successful reattachment of macula-off RRD [17]. These results suggest that the persistent SMF would not significantly affect the recovery of the photoreceptors after successful reattachment.

Animal studies have shown that the photoreceptors under a detached retina undergo apoptosis [30–32]. After the occurrence of a retinal break, the vitreous humor leaks into the subretinal space creating the retinal detachment, and this progresses to apoptosis of the photoreceptors in eyes with a RRD [30–32]. Arroyo et al obtained retinal tissue fragments during RRD surgery, and histological analyses showed that apoptosis occurred within 24 hours after the retinal detachment, peaked on day 2, and decreased to a low level after day 7 of the detachment [32]. Experimental studies have demonstrated a loss of the outer segment of the photoreceptors after a separation of the photoreceptors from the RPE thereby disrupting normal outer segment renewal and leading to a shortening of the outer segments and eventual degeneration of the photoreceptors [33–35].

Taken together, our results indicate that the residual SMF after absorption of the constituents of the vitreous humor in the preoperative SMF would not affect the photoreceptors resulting in an improvement of vision and a recovery of the photoreceptors. In addition, the clinical importance of these finding would support a strategy that it is better to observe the persistent SMF rather than additional procedures including surgery to try to remove the persistent SMF.

There are several limitations to this study. First, this was a retrospective study on a relatively small sample size which would lower the statistical power of the analyses. Second, the postoperative period was relatively short at 12 months although the SMF was absorbed in 12 months in all the cases. Third, we did not collect subretinal fluid during surgery and do not know the characteristics of the SMF. Fourth, the presence of the SMF was judged at the visits, and we could not obtain SD-OCT images at very short intervals. Therefore, it was unclear when the SRF disappeared. Fifth, the retinal layer distances were manually measured because automated calculation of retinal layer thicknesses is difficult to perform in eyes which retinal layers are fragmented and not clearly distinguishable. Further prospective studies on a greater number of cases with evaluation of characteristic of the SMF and automated calculation of retinal thicknesses will be necessary.

In conclusion, the preoperative status of the retina in which retinal break(s) is/are open and the vitreous humor flows into the subretinal space before surgery should influence the final vision, but postoperative residual SMF would not significantly disturb the recovery of the photoreceptors in eyes with macula-off RRD treated by SB surgery.

## Supporting information

S1 Dataset (XLSX)
